# Clinical utilization of genomics data produced by the international *Pseudomonas aeruginosa* consortium

**DOI:** 10.3389/fmicb.2015.01036

**Published:** 2015-09-29

**Authors:** Luca Freschi, Julie Jeukens, Irena Kukavica-Ibrulj, Brian Boyle, Marie-Josée Dupont, Jérôme Laroche, Stéphane Larose, Halim Maaroufi, Joanne L. Fothergill, Matthew Moore, Geoffrey L. Winsor, Shawn D. Aaron, Jean Barbeau, Scott C. Bell, Jane L. Burns, Miguel Camara, André Cantin, Steve J. Charette, Ken Dewar, Éric Déziel, Keith Grimwood, Robert E. W. Hancock, Joe J. Harrison, Stephan Heeb, Lars Jelsbak, Baofeng Jia, Dervla T. Kenna, Timothy J. Kidd, Jens Klockgether, Joseph S. Lam, Iain L. Lamont, Shawn Lewenza, Nick Loman, François Malouin, Jim Manos, Andrew G. McArthur, Josie McKeown, Julie Milot, Hardeep Naghra, Dao Nguyen, Sheldon K. Pereira, Gabriel G. Perron, Jean-Paul Pirnay, Paul B. Rainey, Simon Rousseau, Pedro M. Santos, Anne Stephenson, Véronique Taylor, Jane F. Turton, Nicholas Waglechner, Paul Williams, Sandra W. Thrane, Gerard D. Wright, Fiona S. L. Brinkman, Nicholas P. Tucker, Burkhard Tümmler, Craig Winstanley, Roger C. Levesque

**Affiliations:** ^1^Institute for Integrative and Systems Biology, Université LavalQuebec, QC, Canada; ^2^Institute of Infection and Global Health, University of LiverpoolLiverpool, UK; ^3^Department of Molecular Biology and Biochemistry, Simon Fraser UniversityVancouver, BC, Canada; ^4^Ottawa Hospital Research InstituteOttawa, ON, Canada; ^5^Faculté de Médecine Dentaire, Université de MontréalMontréal, QC, Canada; ^6^QIMR Berghofer Medical Research InstituteBrisbane, QLD, Australia; ^7^Seattle Children's Research Institute, University of Washington School of MedicineSeattle, WA, USA; ^8^School of Life Sciences, University of NottinghamNottingham, UK; ^9^Département de Médecine, Université de SherbrookeSherbrooke, QC, Canada; ^10^Centre de Recherche de l'Institut Universitaire de Cardiologie et de Pneumologie de QuébecQuebec, QC, Canada; ^11^Département de Biochimie, de Microbiologie et de Bio-informatique, Faculté des Sciences et de Génie, Université LavalQuebec, QC, Canada; ^12^Department of Human Genetics, McGill UniversityMontreal, QC, Canada; ^13^INRS Institut Armand FrappierLaval, QC, Canada; ^14^School of Medicine, Griffith UniversityGold Coast, QLD, Australia; ^15^Department of Microbiology and Immunology, University of British ColumbiaVancouver, BC, Canada; ^16^Biological Sciences, University of CalgaryCalgary, AB, Canada; ^17^Department of Systems Biology, Technical University of DenmarkLyngby, Denmark; ^18^M.G. DeGroote Institute for Infectious Disease Research, McMaster UniversityHamilton, ON, Canada; ^19^Antimicrobial Resistance and Healthcare Associated Infections Reference Unit, Public Health EnglandLondon, UK; ^20^Child Health Research Centre, The University of QueenslandBrisbane, QLD, Australia; ^21^Centre for Infection and Immunity, Queen's University BelfastBelfast, UK; ^22^Klinische Forschergruppe, Medizinische HochschuleHannover, Germany; ^23^Department of Molecular and Cellular Biology, University of GuelphGuelph, ON, Canada; ^24^Department of Biochemistry, University of OtagoDunedin, New Zealand; ^25^Institute for Microbiology and Infection, University of BirminghamBirmingham, UK; ^26^Department of Infectious Diseases and Immunology, The University of SydneySydney, NSW, Australia; ^27^Department of Pneumology, Institut Universitaire de Cardiologie et de Pneumologie de Québec, Université LavalQuebec, QC, Canada; ^28^Department of Microbiology and Immunology and Department of Experimental Medicine, McGill UniversityMontreal, QC, Canada; ^29^Department of Biology, Bard College, Annandale-On-HudsonNY, USA; ^30^Laboratory for Molecular and Cellular Technology, Queen Astrid Military HospitalBrussels, Belgium; ^31^New Zealand Institute for Advanced Study, Massey UniversityAlbany, New Zealand; ^32^Max Planck Institute for Evolutionary BiologyPlön, Germany; ^33^Department of Biology, University of MinhoBraga, Portugal; ^34^St. Michael's HospitalToronto, ON, Canada; ^35^Strathclyde Institute of Pharmacy and Biomedical Sciences, University of StrathclydeGlasgow, UK

**Keywords:** *Pseudomonas aeruginosa*, next-generation sequencing, bacterial genome, phylogeny, database, cystic fibrosis, antibiotic resistance, clinical microbiology

## Abstract

The International *Pseudomonas aeruginosa* Consortium is sequencing over 1000 genomes and building an analysis pipeline for the study of *Pseudomonas* genome evolution, antibiotic resistance and virulence genes. Metadata, including genomic and phenotypic data for each isolate of the collection, are available through the International *Pseudomonas* Consortium Database (http://ipcd.ibis.ulaval.ca/). Here, we present our strategy and the results that emerged from the analysis of the first 389 genomes. With as yet unmatched resolution, our results confirm that *P. aeruginosa* strains can be divided into three major groups that are further divided into subgroups, some not previously reported in the literature. We also provide the first snapshot of *P. aeruginosa* strain diversity with respect to antibiotic resistance. Our approach will allow us to draw potential links between environmental strains and those implicated in human and animal infections, understand how patients become infected and how the infection evolves over time as well as identify prognostic markers for better evidence-based decisions on patient care.

## Importance of *P. aeruginosa* as a model in large-scale bacterial genomics

Studies of the genetic structure of microbial populations are central to understand the evolution, ecology and epidemiology of infectious diseases. However, numerous studies describing the genetic structure of pathogen populations are based on samples drawn mostly and overwhelmingly from clinical collections (Wiehlmann et al., 2007; Pirnay et al., 2009). This approach has resulted in a limited view of bacterial pathogens with respect to the evolutionary history of disease-causing lineages as well as the development and distribution of antibiotic resistance genes via the resistome and mobilome (D'Costa et al., 2006; Perry and Wright, 2013). The environmental bacterium and opportunistic pathogen *P. aeruginosa* is a model system in large-scale bacterial genomics (Gellatly and Hancock, 2013). It exhibits extensive metabolic adaptability enabling survival in a wide range of niches including soil, water, plants and animals. Genome rearrangements and a varying complement of genes contribute to strain-specific activities but the detailed molecular mechanisms are still poorly understood (Silby et al., 2011). Multiple studies, some of which include environmental isolates, have sought to resolve the population structure of *P. aeruginosa* using various typing methods and are not in full agreement (Kiewitz and Tümmler, 2000; Pirnay et al., 2002, 2009; Wiehlmann et al., 2007; Fothergill et al., 2010; Lam et al., 2011; Kidd et al., 2012; Martin et al., 2013). Next-generation sequencing (NGS) coupled with whole genome comparison is now becoming the new gold standard for understanding bacterial population structure and offers previously unmatched resolution for phylogenetic analysis (Hilker et al., 2015; Marvig et al., 2015; Williams et al., 2015). The combination of NGS with a more extensive set of strains promises to resolve the population structure of *P. aeruginosa* and shed light on the genetic basis of its adaptability.

## Prominent role of *P. aeruginosa* in cystic fibrosis lung infections

*Pseudomonas aeruginosa* can cause serious opportunistic infections in humans, in particular among immunocompromised individuals, those having cancer, skin burns and wounds, and most notably, cystic fibrosis (CF) patients (Lyczak et al., 2000). Although transmission routes are difficult to establish, it is generally accepted that the lungs of most CF patients become infected with *P. aeruginosa* from the environment, and it is difficult to devise strategies to counter such infections (Emerson et al., 2002; Hauser et al., 2011). Even though patient management has increased the median life expectancy of CF patients to about 50 years (Stephenson et al., 2015), most patients eventually develop chronic lung infection, which is the main cause of morbidity and mortality associated with CF. While there has been significant progress in early eradication therapy for *P. aeruginosa* (Lee, 2009; Heltshe et al., 2015), chronic lung infections remain challenging to eradicate. Indeed, the basic principles on which clinical bacteriology practices are based have altered little over the past 50 years and suffer severe limitations in the context of opportunistic and chronic lung infections. *P. aeruginosa* populations often exist in biofilms and diversify phenotypically in the CF lung (Mowat et al., 2011), hence antimicrobial susceptibility profiles applied to single isolates are poorly predictive of therapeutic efficacy (Keays et al., 2009). Moreover, despite the availability of a number of completely sequenced and annotated *P. aeruginosa* genomes (Stover et al., 2000; Lee et al., 2006; Winstanley et al., 2009; Roy et al., 2010; Jeukens et al., 2014), knowledge on genome evolution and the genomic requirements for opportunistic and CF infections is limited. In fact, sequenced strains reported to date (www.pseudomonas.com, Winsor et al., 2011) were randomly selected and are unlikely to reflect population diversity, hence representing an incomplete snapshot of the pathogen, even in the context of CF. Understanding the biology of *P. aeruginosa* requires the exploration of nontraditional niches in the environment and the widest possible repertoire of opportunistic infections.

## The international *P. aeruginosa* consortium (IPC)

The science of genomics applied to opportunistic infections via whole bacterial genome sequencing promises to transform the practice of clinical microbiology. With rapidly falling costs and turnaround times, microbial genome sequencing and analysis are becoming a viable strategy to understand CF lung infections as well as other human and animal infections. The objective of the International *P. aeruginosa* Consortium (IPC) is to sequence a minimum of 1000 *P. aeruginosa* genomes, link the data to the *Pseudomonas* Genome Database (www.pseudomonas.com, Winsor et al., 2011), integrate the information with the Canadian CF registry and develop a user-friendly pipeline to study these genomes. Genomics data will support molecular epidemiology for the surveillance of outbreaks and has the potential for future genotypic antimicrobial susceptibility testing as well as the identification of novel therapeutic targets and prognostic markers. This project is supported by an international consortium from five continents; its outcomes will have worldwide dissemination for the benefit of clinical microbiology and especially for CF patients.

## Sequencing over 1000 *P. aeruginosa* genomes: objectives and strategy

By generating a comprehensive genome sequence database truly representative of the worldwide *P. aeruginosa* population, we will:

1. Assemble a large and representative strain collection, with associated genome data, useful for antimicrobial testing, identification of resistance markers, and data mining for new therapeutic targets;
2. Develop platforms and pipelines to enable synergy between genomic and clinical data, which will allow identification of prognostic markers and stratification of patients, leading to improvements in patient care;
3. Transform CF diagnostic microbiology through innovations in genomics;
4. Develop user-friendly tools that will enable CF clinicians to interpret genomic data leading to better informed decisions on issues of cross infection.

Our working hypothesis is that a high-quality, large-scale bacterial genome database available through a user-friendly pipeline will have a major impact on epidemiology, diagnostics and treatment. A major objective is to identify representative isolates from groups of closely related genomes to become reference type isolates and provide reference type genomic data. To this end, genomes are initially sequenced on an Illumina MiSeq instrument with an average median coverage of 40. This first sequencing step will help to determine whether there is already a good reference genome for each new genome by investigating both core and accessory genetic material. First, for conserved regions among isolates, i.e., the core genome, we will perform phylogenetic analysis and identify single nucleotide polymorphisms (SNPs). Second, we will determine whether a new genome significantly contributes to expansion of the full genetic repertoire of *P. aeruginosa* (i.e., the pan-genome), for instance, with at least 0.1% of its genome (about 6000 bp) representing previously unknown accessory genetic material. It will also be possible to identify indels and structural variations among genomes. We will analyze genomes for the presence of genomic islands using IslandViewer and related genome-comparison and sequence-composition based tools (Langille and Brinkman, 2009; Grant et al., 2012; Dhillon et al., 2015). Finally, each new genome will be characterized based on the presence/absence of regions of genome plasticity (Klockgether et al., 2011), the virulence factor (VF) database (Barken et al., 2008), curated VF data at www.pseudomonas.com, and the Comprehensive Antibiotic Resistance Database (McArthur et al., 2013). In light of this information, a limited set of new reference genomes will eventually be selected for PACBIO RS II sequencing to enable full assembly and detailed annotation.

## The international *P. aeruginosa* consortium database (IPCD)

The IPC's strain collection is harbored at the Institute for integrative and systems biology (IBIS), in Quebec City, Canada. It currently contains 1514 entries for *P. aeruginosa* isolates spanning 135 years back to 1880 and covering 85 locations, in 35 countries, on five continents. It includes previously described collections (Pirnay et al., 2009; Stewart et al., 2011; Kidd et al., 2012) and was assembled with the aim of representing maximal genomic diversity. To this end, various criteria were taken into consideration, including geographic origin, previous genotyping, phenotype, and *in vivo* behavior. We envisage that the collection can accommodate over 10,000 isolates.

In order to manage phenotypic and genomic data for the growing *P. aeruginosa* collection in addition to sharing this data, we created the International *P. aeruginosa* Consortium Database (IPCD), an open source web application available at http://ipcd.ibis.ulaval.ca/. It includes isolate identification, host, researcher, date of isolation, geographical origin, phenotypic data, anonymized patient information, DNA extraction details, NGS information and genome assembly. Its structured vocabulary is being developed further. IPCD currently contains NGS data and unpublished draft genomes from CF patients and from most of the other types of known human infections. For comparative genomics purposes, IPCD also contains animal infection isolates and environmental isolates from plants, soil and water. Researchers who provided strains have priority access to corresponding genome sequences through personal user accounts, but all genome sequences produced by the IPC will become publically available.

## The IBIS bioinformatics pipeline for bacterial genome assembly

Analysis software for genome assembly and selection of additional reference genomes is required to extract relevant information in a fully automated and reliable fashion without human intervention. Ideally, this software should be platform independent and analyze sequence data directly without being tied to proprietary data formats. This ensures maximal flexibility and reduces lag time to a minimum. We are currently using an integrated pipeline for *de novo* assembly of microbial genomes based on the A5 pipeline (Tritt et al., 2012) and parallelized on a Silicon Graphics UV 100 to accommodate data from 96 genomes and provide assembly statistics in about 30 h. This automated approach currently results in 20–60 contigs per genome (median N50 = 415 kb) and is anticipated to improve as sequencing technology improves.

## Phylogeny of *P. aeruginosa*

*P. aeruginosa* is well known to have an adaptable genome (5.5–7 Mbps) that enables it to colonize a wide range of ecological niches; comparative genomics approaches have identified changes in surface antigens, loss of virulence-associated traits, increased antibiotic resistance, inserted genomic islands including phage, and pyocin operons, overproduction of alginate and the modulation of metabolic pathways. Its genome also has many regions that exhibit plasticity (Klockgether et al., 2011). The IPC will provide fine-scale analyses to evaluate these changes, allowing the comparison of VFs, loss/gain of function mutations and antibiotic resistance genes as well as complete core and accessory genomes.

**Figure 1:**
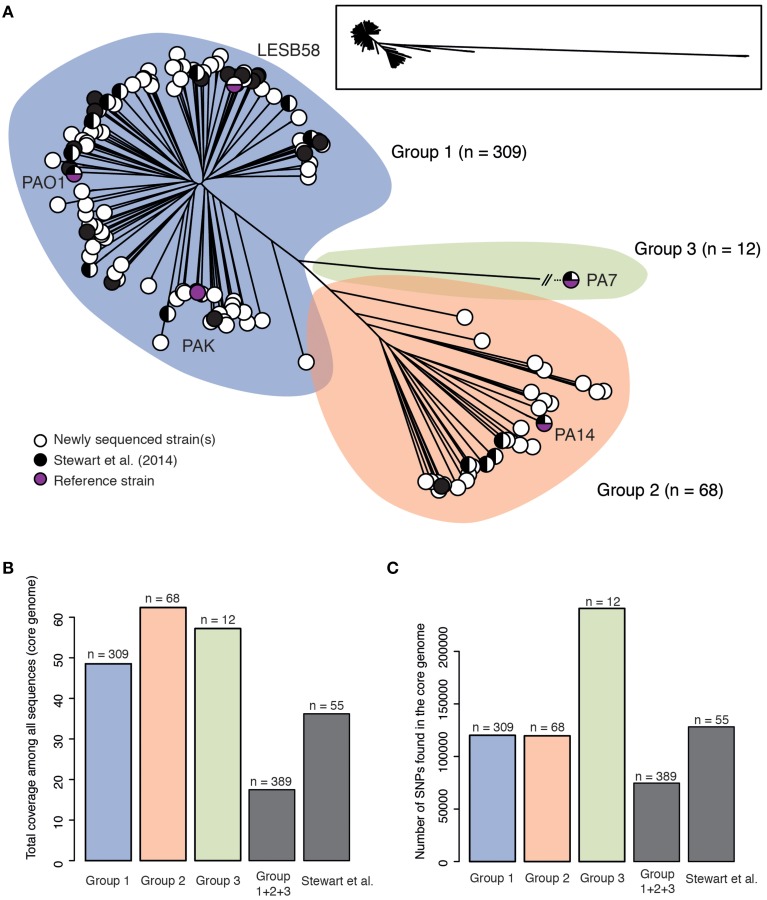
**(A)** Unrooted maximum likelihood tree of 389 *Pseudomonas aeruginosa* genomes based on SNPs within the core genome as defined by Harvest (100 bootstraps). Strains are divided into three major groups (group 1: blue, group 2: orange and group 3: green). The number of strains for each group is shown. Black circles represent strains that were already sequenced before this study while white circles represent one or more strains that were sequenced in this study. Group 3 was contracted for visualization purposes; a framed miniature of the true appearance of this tree is presented. The tree in Newick format is available as Supplementary Data Sheet 1 (B) Total coverage of the *P. aeruginosa* genome by the core genome for each of the three groups shown in **(A)**, all 389 genomes (Group 1+2 + 3) and a diverse set of 55 strains from Stewart et al. (2014). **(C)** Total number of core genome SNPs for each of the three groups shown in **(A)**, all 389 genomes (Group 1+2 + 3) and a diverse set of 55 strains from Stewart et al. (2014).

As a proof of concept for this aim, we used the 389 genomes that constitute our current sequence dataset, including the 335 draft genomes produced to date by the IPC, to perform phylogenetic analysis of the core genome using the Harvest suite (Treangen et al., 2014). We found that *P. aeruginosa* strains can be divided into three major groups (Figure 1A), a result providing concrete support and agreement to previous results with a limited but diverse set of 55 strains (Stewart et al., 2014). The tree presented here also provides novel information since it shows new subgroups and provides unmatched resolution. The high number of strains included in our analysis and the fact that these strains come from a wide array of sources (including environmental, clinical and animal strains, and a wide geographical spread) may suggest that group 1 strains, including PAO1, are naturally more abundant than group 2, which includes PA14. However, it is noteworthy that the opposite conclusion was reached in a previous typing-based study (Wiehlmann et al., 2007), and that our dataset is biased toward clinical isolates. Further, the third major group that includes strain PA7 is now populated with 12 new strains. Given that phylogenetic analysis of 389 genomes has produced at least 20 distinct branches, it is clear that a more extensive survey sequencing approach, as proposed by the IPC, is required to populate these branches. Data analysis with Harvest also revealed that the core genome represents 17.5% of the average *P. aeruginosa* genome size. This is much less than what previous studies, which typically do not include group 3 strains, have reported (e.g., 79% in Dettman et al., 2015), and is due to a combined effect of diversity and number of strains, as it can be deduced from Figure 1B. Inclusion of sister species *P. resinovorans* in this analysis resulted in a drop of core genome coverage to 2.4%. Figure 1C presents the number of core genome SNPs.

## Linking IPCD with pseudomonas.com and the comprehensive antibiotic resistance database

The *Pseudomonas* Genome Database (www.pseudomonas.com; Winsor et al., 2011) is an established platform for searching and comparing multiple genome sequences and annotations for *Pseudomonas* species. This publically available database hosts sequence and annotation data including orthologs, function, expression, cross references, and various predictions for 70 complete *Pseudomonas* genomes plus draft genomes for more than 561 additional *P. aeruginosa* isolates. It provides ongoing high-quality curated updates to existing annotations based on community involvement. The IPCD will draw on existing tools such as Sybil that have been developed for both carrying out comparative analyses and presenting data over the web (Riley et al., 2012). Reliable methods for the phylogenetic analysis of our dataset are used including analysis of core genome SNPs using Harvest (Treangen et al., 2014). Close attention to the links between the presence of strain specific genomic islands and patterns of SNPs in the core genome will help identify diagnostic sequences and SNP combinations for the development of new *P. aeruginosa* typing methods with the highest resolution to date. This will be done using a combination of *de novo* island prediction using IslandViewer (Langille and Brinkman, 2009; Dhillon et al., 2015) and analysis using the CG View Comparison Tool (Grant et al., 2012).

**Figure 2:**
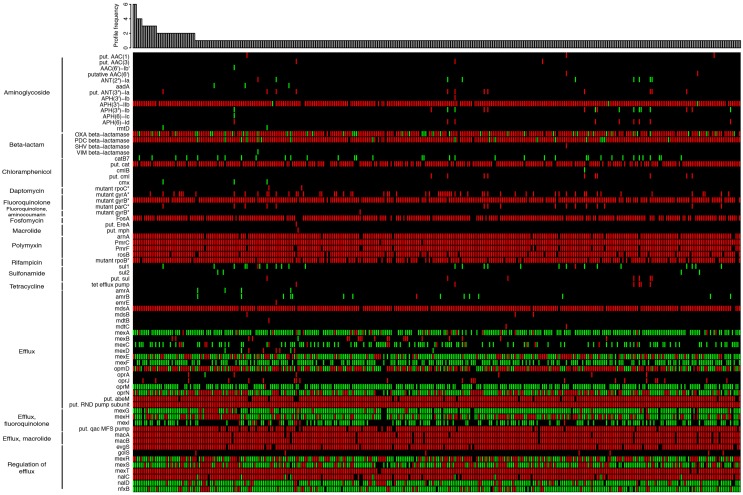
**Heat map showing the unique distribution profiles of antibiotic resistance genes for 389 ***Pseudomonas aeruginosa*** strains (black: no sequence matching the protein; green: perfect match to known antimicrobial resistance (AMR) gene sequence; red: variant of known AMR gene sequence)**. The heat map was obtained by performing a Resistance Gene Identifier (RGI) analysis against reference sequences of the Comprehensive Antibiotic Resistance Database (CARD; McArthur et al., 2013). The bar plot shows in how many strains each profile was observed. On the left, proteins are grouped according to their biological function or the resistance they confer. In rare cases, more than a single copy of a resistance gene may be present within an individual strain. For those genes with resistance conferred by mutation (labeled with an asterisk), all detected mutations are known from other pathogens and may require functional verification in *P. aeruginosa*. Genes labeled as “putative” (“put.” in the figure) are similar to a number of known sequence variants within a family of AMR genes. All perfect matches to OXA β-lactamases are OXA-50. The complete heat map with the full set of *P. aeruginosa* strains is available in Supplementary Image 1. The raw data used to generate the heat map is available as Supplementary Table 1.

As an additional feature, we plan to link IPCD with The Comprehensive Antibiotic Resistance Database (CARD; McArthur et al., 2013) available at http://arpcard.mcmaster.ca/, which provides data for ~3000 antibiotic resistance genes and is under continuous curation efforts. Here, for instance, we used the CARD reference data to detect the presence of resistance genes through Resistance Gene Identifer searches (McArthur et al., 2013) on 389 *P. aeruginosa* strains (Figure 2). Approximately 40% of the 73 detected resistance genes were found in a majority of strains, including genes involved in transport, efflux and permeability as well as genes involved in beta-lactam resistance. Approximately 60% of the resistance genes we detected were found only in a restricted group of strains, particularly for aminoglycoside, macrolide, and sulfonamide resistance, highlighting the great variability of *P. aeruginosa* strains with respect to resistance genes. This variation is now being unraveled thanks to our extensive sampling. These data will be used to study and understand the pool of resistance genes present in clinical strains with a particular focus on CF strains, and to understand the links between clinical and environmental strains with respect to these genes.

## Linking genomic and clinical data

It will be essential to match phenotypic and clinical data (antibiotic resistance, virulence, anonymized clinical observations) to the genomic data produced. We will categorize data within the IPCD so that isolates can be sorted by phenotype, allowing rapid identification of linked genomic signatures and the development of prognostic approaches to treat CF infections. The development of a pipeline to initially map “new” *P. aeruginosa* genomes will evolve toward becoming a routine clinical tool for the use of genomic data in CF and could represent a very powerful sentinel surveillance system. We will develop tools to rapidly collate data for a given strain type and produce a concise phenotypic and clinical profile that provides clinicians with an evidence based decision making platform. The Canadian CF Registry was created so that data entry could be standardized in CF clinics across Canada. We will use this system to link IPCD with clinical data for CF isolates.

## Future genomic analyses and biological studies of *P. aeruginosa*

We are committed to continuously improve the IPCD and pseudomonas.com by adding *P. aeruginosa* genomes from other human, animal, and environmental isolates as well as by enhancing metadata. The consortium has identified a number of research priorities in the international *P. aeruginosa* community, many of which will be long-term projects shared among members of the consortium as a research working group. The goal of the IPC is to avoid duplication of efforts in *P. aeruginosa* genomics and enhance interest from researchers having common goals. Additional members are welcome to join in so that the “depth and breadth” of *P. aeruginosa* biology expands beyond what we outlined for the initial consortium. We also intend to seek collaboration with other groups to connect our database with those developed for other Pseudomonad genomes. Finally, the IPC could become a model for other groups interested in the bacterial genomics of infectious diseases, as the combination of large-scale genomics and evolutionary biology tools may lead to new strategies for countering infections (Little et al., 2012; Casadevall and Pirofski, 2014).

## Author contributions

LF, JJ, IK, and RL collected *Pseudomonas* strains, performed the analyses and drafted the manuscript. BB provided support for sequencing and analysis. MD, JL, SL, and HM contributed to the genome assembly pipeline and the development of IPCD. AM, BJ, SP, and NW performed the resistome analysis. GW and FB provided database input for links with www.pseudomonas.com. All other authors handled *Pseudomonas* strains and collected metadata. All authors revised the manuscript.

### Conflict of interest statement

The authors declare that the research was conducted in the absence of any commercial or financial relationships that could be construed as a potential conflict of interest.

## Supplementary material

Supplementary Table 1 **Complete antibiotic resistance proteins matrix**.

Supplementary Data Sheet 1 **Core genome phylogenetic tree (FigTree)**.

Supplementary Image 1 **Complete antibiotic resistance proteins heat map**.
